# Effect of various organic acid supplementation diets on *Clarias gariepinus* BURCHELL, 1822: Evaluation of growth, survival and feed utilization

**DOI:** 10.12688/f1000research.15954.1

**Published:** 2018-09-14

**Authors:** Lia Asriqah, Rudy Agung Nugroho, Retno Aryani

**Affiliations:** 1 Animal Physiology, Development, and Molecular Laboratory, Department of Biology, Faculty of Mathematics and Natural Sciences, Mulawarman University, Samarinda, Kalimantan Timur, 75123, Indonesia; 2 Animal anatomy and Microtechnique Laboratory, Department of Biology, Faculty of Mathematics and Natural Sciences, Mulawarman University, Samarinda, Kalimantan Timur, 75123, Indonesia

**Keywords:** Organic acid, Growth, Survival Rate, Feed utilities, Clarias gariepinus

## Abstract

**Background:** The purpose of the current study was to determine the growth status, survival and feed utilization of catfish ( *Clarias gariepinus* BURCHELL, 1822) fed various organic acid supplementations.

**Methods:** In total, 1600 fish were randomly distributed into 20 tanks and fed different types of diet: A, control diet without organic acid supplementation; B, control diet supplemented with 0.05% formic, acetic, and propionic acid; C, control diet supplemented with 0.1% formic, acetic, and propionic acid; D, control diet supplemented with 0.05% butyric acid; E, control diet supplemented with 0.01% butyric acid. The control diet was a commercial diet, containing 35% crude protein, 8.58% crude fat, and 2.75% fibre. All fish were fed using a satiation method, three times per day for 56 days. At the end of the trial, growth, survival and feed utilization were determined. Water quality parameters during the trial were also measured once a week.

**Results:** Fish fed diet type D had the significantly lowest ( *P<0.05*) final weight (FW), weight gain (WG), and specific growth rate (SGR) of all diets. Similar FW, WG, and SGR were found for fish fed diets A-C and E. Meanwhile, the feed conversion ratio, feed efficiency, and survival rate of fish were not affected by any types of diet. The water quality parameters were not significantly different between tanks and weeks: dissolved oxygen 6.79-6.81 mg L ^-1^, pH 7.11-7.19, water temperature 28.97-29.32°C, nitrite (NO _2_) content 0.48- 0.50 mg L ^-1^, and ammonia (NH _3_) content 0.064-0.066 mg L ^-1^.

**Conclusion:** The supplementation of 0.05% butyric acid in the diet of *C. gariepinus* for 56 days reduced the growth performance of the fish. However, supplementation of an organic acid in the diet of *C. gariepinus* had no impact on feed utilization, survival, and water quality parameters.

## Introduction

Optimum and balanced nutrition, especially in fish culture, is a significant requirement and contributes up to 40–60% of production cost of farmed fish ^1, 2^. The balance of a commercial diet that enhances optimum fish growth and health has attracted much research to develop a specific diet formulation ^1^. It is also well known that the use of antibiotics or chemical substances as a growth promoter in the feed of fish may help to improve growth, survival, and feed utilization. However, wider concerns regarding the negative effects to the environment has led to a ban of the use of such chemical substances in the field of aquaculture ^3^.

Previous research stated that the use of non-chemical substances, such as acidifiers, to increase growth performance has been performed in several fish. Dietary supplementation of citric acid/formic acid increases the bioavailability of minerals, including phosphorus, magnesium, calcium and iron in rainbow trout ( *Oncorhynchus mykiss*), sea bream ( *Pagrus major*) and Indian carp ( *Labeo rohita*) ^4, 5^. Some researchers also claimed that dietary acidifiers in the feed of fish reduce the pH in the stomach and foregut, which help improve pepsin activity, enhancing protein metabolism and mineral intake of the intestines ^4, 6^. In addition, these short-chain organic acids are generally absorbed through the intestinal epithelia by passive diffusion, providing energy for renewing the intestinal epithelia and maintaining gut health ^7^.

Besides nutritional concern in aquafeed, generally aquaculture activities commonly produce waste, such as feed remains and feces, which can be converted into ammonia and nitrite. Further, the level of ammonia (NH _3_) and nitrite (NO _2_) increases rapidly in a closed culture system and can be harmful to fish ^8, 9^. Thus, water quality parameters are a major concern in the aquaculture system. Previous research revealed that the values of water quality parameters during fumaric acid feeding experiments on the African catfish ( *Clarias gariepinus*) are relatively stable, providing a dissolved oxygen concentration 7.23-7.86 mg L ^-1^, water temperature 25.13-25.27 ^0^C and pH 7.23-7.48 ^10^.

A strain of African catfish, *Clarias gariepinus* BURCHELL, 1822, is a popular species for aquaculture industry in Asian countries. In Indonesia, the production of catfish is been the second largest after tilapia, reaching a production from 144,755 MT in 2009 to 644,221 MT in 2013 ^11^. Catfish has pseudo-lungs, long bodies and a high capacity to produce mucous as a form of adaptation to live in stagnant environments or drought conditions. It is omnivorous, feeding on various feeds, such as plant material, plankton, arthropods, molluscs, fish, reptiles, and amphibians ^12^. Compared to other species, catfish is more resistance to diseases, handles stressors well and has a high growth performance ^13^. To increase growth performance, aquaculturists and researchers have added various supplementations to the diet of catfish ^14– 16^. However, the information regarding supplementation of organic acid (formic, acetic, propionic and butyric acid) in the diet of catfish is very rare. Thus, the aim of the current experiment was to evaluate the growth performance, feed utilization, and survival of catfish fed different types of diet, containing organic acid.

## Methods

### Site and time

The research was performed at PT Suri Tani Pemuka *Unit Research and Development*, Ciranjang, West Java, Indonesia from March to May 2018. All *C. garipienus* were provided by PT Suri Tani Pemuka (Cisarua, Tegal Waru, HIAT Purwakarta Regency, West Java, Indonesia). The fish were kept in oxygenated polythene bags and transported by truck to PT Suri Tani Pemuka, Research and Development Farm, Ciranjang West Java, Indonesia. Then, the fish had been adapted and grown under farming conditions.

The study was carried out within The PT Suri Tani ethical protocols of the farm. 

### Experimental design

Five groups in five separate tanks, namely: A, control diet without organic acid supplementation; B, control diet supplemented with 0.05% formic, acetic, and propionic acid; C, control diet supplemented with 0.1% formic, acetic, and propionic acid; D, control diet supplemented with 0.05% butyric acid; E, control diet supplemented with 0.01% butyric acid. The control diet which was provided from a commercial diet (provided by PT Suri Tani Pemuka, Purwakarta, West Java, Indonesia), containing 35% crude protein, 8.58% crude fat, and 2.75% fibre. The study was repeated four times. All fish were maintained in a plastic tank (vol. 520 L) at a stocking density of 80 fish per tank and reared for 56 days.

### Fish culture and feeding trial

In total 1600 fish with an initial average weight 8.78 g were randomly assigned into 20 plastic tanks (80 fish/tank) with a volume of 520 L. Each tank was filled with fresh water up to 500 L and the fish were stocked at the density of 80 fish tank ^-1^. The fish were fed with diets A–E three times per day (01:00, 05:00 and 09:00 GMT) using satiation methods for 56 days. 

### Measured parameters

Biomass (g) of the fish per tank were measured at the beginning and the final day of the study. Meanwhile, the weight gain was calculated using equation: 

      W = (Wt/Nt)-(W0/N0)

where W is weight gain (g), Wt is the weight of the fish at the end of trial (g), and Wo is the weight of fish at the beginning of the trial (g). The feed utilization and survival rates were determined following equations that were previously used by Muchlisin ^17^ and Nugroho ^18^:

      Feed efficiency (FE) = 1/ FCR × 100%

where FCR = feed conversion ratio:

      FCR = F / (Wt – Wo)

where F= total feed intake (g).

      Survival rate (SR) = (Nt/N0) × 100%

where Nt is total fish at the end of experiment and N0 is total fish at start of experiment.

The water quality parameters such as dissolved oxygen (DO) and temperature were measured using a digital water checker (YSI™ Model 550A Dissolved Oxygen Meter; Fisher Scientific, USA). pH was measured with a pH-meter (CyberScan pH 11; EuTech Instruments, Singapore). Meanwhile, NO _2_ and NH _3_ were detected using Sera test kit (Sera GmbH D52518, Heinsberg, Germany). All the water quality parameters were measured once a week.

### Data analysis

Results are expressed as means ± standard error (SE) and data were analysed using SPSS version 22 (SPSS, Inc., USA). The data of survival (%) was transformed using arc sine before statistical analysis. Meanwhile, growth analysis and water quality were subjected to analysis of variance (ANOVA), followed by Duncan post hoc test to evaluate significant differences among the groups of treatments. All significant tests were at *P>*0.05.

## Results

Based on the statistical analysis, the present results showed that both the control diet (A) and the supplementation organic acid in the diet of *Clarias gariepinus* (B–E) had no significant effect ( *P>0.05*) on the feed conversion ratio (FCR), feed efficiency (FE), and survival rate (SR). The trial also showed that fish fed diet D had the significantly lowest ( *P<0.05*) final weight, weight gain, and specific growth rate (SGR), but a similar final weight, weight gain, and SGR were found on fish fed diets A–C, and E ( Table 1).

**Table 1.** Mean ± SE growth and feed utilities of *Clarias gariepinus* BURCHELL, 1822 fed organic acid supplementation in the diet for 56 days.

Parameters	Diets				
	A	B	C	D	E
Initial weight (g)	702.50±2.50 ^a^	702.50±2.50 ^a^	702.50±2.50 ^a^	702.50±2.50 ^a^	700.00±2.50 ^a^
Final weight (g)	7065.00±136.16 ^ab^	7007.50±161.10 ^ab^	7307.50±199.30 ^a^	6702.50±206.37 ^b^	7205.00±163.01 ^ab^
Weight gain (g)	87.07±2.09 ^a^	84.42±2.59 ^ab^	87.09±3.06 ^a^	78.78±1.78 ^b^	87.96±2.11 ^a^
SGR (% day ^-1^)	4.26±0.04 ^a^	2.21±0.05 ^ab^	4.26±0.05 ^a^	4.10±0.03 ^b^	4.29±0.03 ^a^
FCR	1.16±0.03 ^a^	1.14±0.03 ^a^	1.11±0.03 ^a^	1.15±0.02 ^a^	1.12±0.02 ^a^
FE (%)	86.41±2.23 ^a^	87.71±2.30 ^a^	90.20±2.74 ^a^	86.73±1.45 ^a^	89.25±1.95 ^a^
SR (%)	92.18±1.72 ^a^	94.06±1.72 ^a^	95.31±0.59 ^a^	95.62±1.19 ^a^	93.12±0.36 ^a^

Different alphabets (a, b) indicate significantly different means for different group of diets at *P < 0.05*. A = control diet without organic acid supplementation; B = supplemented-control diets with 0.05% mix of formic, acetic, and propionic acid; C = supplemented-control diets with 0.1% mix of formic, acetic, and propionic acid; D = supplemented-control diets with 0.05 % of butyric acid; E = supplemented-control diets with 0.1% of butyric acid; SGR = Specific growth rate, FCR = Feed conversion ratio, FE = Feed efficiency, SR = Survival rate. The control diet was a commercial diet, containing 35% crude protein, 8.58% crude fat, and 2.75% fibre.

The water quality parameters during the study showed that the supplementation organic acid in the diet of *Clarias gariepinus* had no effects on the water quality culture. Dissolved oxygen ranged 6.81–6.88 mg L ^-1^; pH 7.12–7.21, and water temperature 27.07–29.50°C. Meanwhile, nitrite (NH _2_) content ranged from 0.045 to 0.057 mg L ^-1^ and the ammonia (NH _3_) content ranged from 0.372 to 0.50 mg L ^-1^ ( Table 2).

**Table 2.** Mean ± SE water quality parameters the cultured media of *Clarias gariepinus* BURCHELL, 1822 during the trial.

Parameters	Diets				
	A	B	C	D	E
Dissolved oxygen (mg L ^-1^)	6.81±0.01 ^a^	6.81±0.01 ^a^	6.78±0.01 ^a^	6.81±0.01 ^a^	6.79±0.01 ^a^
pH	7.17±0.04 ^a^	7.21±0.05 ^a^	7.06±0.04 ^a^	7.11±0.04 ^a^	7.19±0.04 ^a^
Temperature (°C)	29.33±0.21 ^a^	29.53±0.20 ^a^	28.71±0.22 ^a^	29.05±0.26 ^a^	29.11±0.18 ^a^
NH _3_ (mg L ^-1^)	0.06±0.001 ^a^	0.06±0.001 ^a^	0.06±0.001 ^a^	0.06±0.001 ^a^	0.06±0.001 ^a^
NO _2_ (mg L ^-1^)	0.46±0.02 ^a^	0.48±0.01 ^a^	0.50±0.00 ^a^	0.50±0.00 ^a^	0.50±0.00 ^a^

Mean ± SE followed by same superscript letter (a) indicated not significantly different at *P < 0.05*. Water quality parameters were measured once a week during the study. A = control diet without organic acid supplementation; B = supplemented-control diets with 0.05% mix of formic, acetic, and propionic acid; C = supplemented-control diets with 0.1% mix of formic, acetic, and propionic acid; D = supplemented-control diets with 0.05 % of butyric acid; E = supplemented-control diets with 0.1% of butyric acid. The control diet was a commercial diet, containing 35% crude protein, 8.58% crude fat, and 2.75% fibre.

The data showing the growth parameters such as initial and final weight, total weight gain, and total feed consumed by fish for every experimental group, and water quality parameters can be seen in Dataset 1.

The initial and final weight, body weight gain, survival, and total feed consumed by fish for every experimental group (A–E) and water quality parameters Click here for additional data file. Copyright: © 2018 Asriqah L et al. 2018 Data associated with the article are available under the terms of the Creative Commons Zero "No rights reserved" data waiver (CC0 1.0 Public domain dedication).

## Discussion

The present results revealed supplementation of organic acid in the diets had no significant effect ( *P>0.05*) on the feed conversion ratio (FCR), feed efficiency (FE), and survival rate (SR). However, dietary supplementation of 0.05% of butyric acid (D) in the diet of *C. gariepinus* resulted in a significantly lower ( *P<0.05*) final weight, weight gain, and SGR compared with other diets. A similar final weight, weight gain, and SGR were also found for fish fed a control diet (A), and those fed with 0.05% (B) and 0.1% (C) mix of formic, acetic, and propionic acid, and 0.1% (E) of butyric acid. These findings are in line with a previous study where dietary 0.5 g kg ^-1^ butyric acid supplementation in the diet of *Clarias gariepinus* found no significant difference in weight gain, SGR, SR, and FCR. In contrast, weight gain, SGR, SR, FE and FCR of *Oreochromis niloticus* were significantly improved after being fed 0.5 g kg ^-1^ butyric acid supplementation in the diet ^16^.

According to Da Silva *et al*. ^19^, butyrate acid in shrimp diets could be feed attractants for fish, which improve feed intake. Organic acids such as butyric acid improve the feed intake, gut and gastrointestinal tract activity of a red hybrid tilapia, *Oreochromis* sp., by the reduction in pH ^3, 20^. The other benefits of butyric acid for improving growth is attributed to the aroma which acts as an attractant in the diet of shrimp ^21^. However, a past study found that the increasing levels of dietary organic acid such as fumaric acid (1.5–2 g kg ^-1^) in the diet of *C. gariepinus* significantly reduced growth performance and feed utilities and improved survival rate after a challenge test with bacteria ^10^. These findings might be correlated with pH balance in the gut of the fish fed dietary high levels of organic acid. Furthermore, various concentrations of the organic acids such as propionic acid and acetic acid, have been determined to have effects on the feeding behaviour of *Oreochromis niloticus*. The supplementation of propionic acid at 10 ^-4^–10 ^-6^ M can stimulate feeding ^22^. However, dietary propionic acid at 10 ^-3^ M may suppress feeding. In addition, past research has also found that dietary supplementation of acetic acid at 10 ^-5^ M had no effect on fish feeding. Lim *et al*. ^23^ revealed that the beneficial of the organic acid supplementation in the diet of fish may vary among fish and tend to be inconsistent, depend on the dietary ingredient, culture system, and water quality.

It is clear that feed remains in the water medium might change the water quality. Current findings stated that the water quality parameters during the trials showed no effects on the medium fish culture during the present study ( Table 2). These findings are consistent with a past study by Omosowone and Adeparusi ^16^, stating that water quality parameters such as temperature, dissolved oxygen and pH measured in a similar current experimental setups are all within the accepted range for the culture of fin fishes in tropical regions, as recommended by National Research Council (USA) ^24^.

## Conclusion

The inclusion of organic acid in the diet of *C. gariepinus* had no impact on the feed utilities, survival, and water quality parameters in the present study. However, the inclusion of 0.05% butyric acid in the diet of *C. gariepinus* for 56 days reduced growth performance and feed utilization. Further research needs to be conducted to evaluate the effects of organic acid supplementation in the diet of fish on digestive enzyme activity, gut bacteria population, and fillet proximate analysis.

## Data availability

The data referenced by this article are under copyright with the following copyright statement: Copyright: © 2018 Asriqah L et al.

Data associated with the article are available under the terms of the Creative Commons Zero "No rights reserved" data waiver (CC0 1.0 Public domain dedication). 

Dataset 1: The initial and final weight, body weight gain, survival, and total feed consumed by fish for every experimental group (A–E) and water quality parameters. DOI, 10.5256/f1000research.15954.d216486 ^25^.

## References

[1] Craig S Helfrich LA Kuhn D: Understanding fish nutrition, feeds, and feeding.2017 Reference Source

[2] Fadri S Muchlisin Z Sugito S: Growth performance, survival rate and feed utilization of Nile tilapia, *Oreochromis niloticus* fed experimental diet contains jaloh leafs, *Salix tetrasperma* Roxb at different levels of EM-4 probiotic. *Jurnal Ilmiah Mahasiswa Kelautan dan Perikanan Unsyiah.* 2016;1(2):210–221.

[3] Luckstadt C: The use of acidifiers in fish nutrition. *Perspectives in Agriculture, Veterinary Science, Nutrition and Natural Resources.* 2008;3(044):1–8. 10.1079/PAVSNNR20083044 PMC8580373 34765015

[4] Jun-sheng L Jian-lin L Ting-ting W: Ontogeny of protease, amylase and lipase in the alimentary tract of hybrid juvenile tilapia ( *Oreochromis niloticus* × *Oreochromis aureus*). *Fish Physiol Biochem.* 2006;32(4):295–303. 10.1007/s10695-006-9106-5

[5] Vielma J Lall S: Dietary formic acid enhances apparent digestibility of minerals in rainbow trout, *Oncorhynchus mykiss* (Walbaum). *Aquac Nutr.* 1997;3(4):265–268. 10.1111/j.1365-2095.1997.00041.x

[6] Lückstädt C: Effect of organic acid containing additives in worldwide aquaculture–sustainable production the non-antibiotic way. *Acidifiers Anim Nutr.* 2008;71.

[7] Abu Elala NM Ragaa NM: Eubiotic effect of a dietary acidifier (potassium diformate) on the health status of cultured *Oreochromis niloticus*. *J Adv Res.* 2015;6(4):621–629. 10.1016/j.jare.2014.02.008 26199753 PMC4506962

[8] Sakala ME Musuka CG: The effect of ammonia on growth and survival rate of tilapia rendalli in quail manured tanks. *International Journal of Aquaculture.* 2014;4.

[9] Sidik A: The effect of stocking density on nitrification rate in a closed recirculating culture system. *Jurnal Akuakultur Indonesia.* 2007;1(2):47–52. 10.19027/jai.1.47-52

[10] Omosowone O Dada A Adeparusi E: Effects of dietary supplementation of fumaric acid on growth performance of African catfish *Clarias gariepinus* and *Aeromonas sobria* challenge. *Croatian Journal of Fisheries.* 2015;73(1):13–19. 10.14798/73.1.782

[11] Fauji H Budiardi T Ekasari J: Growth performance and robustness of African Catfish *Clarias gariepinus* (Burchell) in biofloc‐based nursery production with different stocking densities. *Aquac Res.* 2018;49(3):1339–1346. 10.1111/are.13595

[12] Vitule JR Umbria S Aranha J: Introduction of the African catfish *Clarias gariepinus* (BURCHELL, 1822) into Southern Brazil. *Biol Invasions.* 2006;8(4):677 10.1007/s10530-005-2535-8

[13] Putra I Rusliadi R Fauzi M: Growth performance and feed utilization of African catfish *Clarias gariepinus* fed a commercial diet and reared in the biofloc system enhanced with probiotic [version 1; referees: 2 approved]. *F1000Res.* 2017;6: 1545. 10.12688/f1000research.12438.1 28944046 PMC5585874

[14] Chris UO Singh N Agarwal A: Nanoparticles as feed supplement on Growth behaviour of Cultured Catfish ( *Clarias gariepinus*) fingerlings. *Materials Today: Proceedings.* 2018;5(3):9076–9081. 10.1016/j.matpr.2017.10.023

[15] El-Husseiny OM Hassan MI El-Haroun ER: Utilization of poultry by-product meal supplemented with L-lysine as fish meal replacer in the diet of African catfish *Clarias gariepinus* (Burchell, 1822). *Journal of Applied Aquaculture.* 2018;30(1):63–75. 10.1080/10454438.2017.1412844

[16] Omosowone O Dada A Adeparusi E: Comparison of dietary butyric acid supplementation effect on growth performance and body composition of *Clarias gariepinus* and *Oreochromis niloticus* fingerlings. *Iranian Journal of Fisheries Sciences.* 2018;17(2):403–412. 10.22092/IJFS.2018.115901

[17] Muchlisin ZA Arisa AA Muhammadar AA: Growth performance and feed utilization of keureling ( *Tor tambra*) fingerlings fed a formulated diet with different doses of vitamin E (alpha-tocopherol). *Archives of Polish Fisheries.* 2016;24(1):47–52. 10.1515/aopf-2016-0005

[18] Nugroho RA Manurung H Nur FM: *Terminalia catappa* L. extract improves survival, hematological profile and resistance to *Aeromonas hydrophila* in *Betta* sp. *Archives of Polish Fisheries.* 2017;25(2):103–115. 10.1515/aopf-2017-0010

[19] da Silva BC do Nascimento Vieira F Mouriño JLP: Salts of organic acids selection by multiple characteristics for marine shrimp nutrition. *Aquaculture.* 2013;384–387:104–110. 10.1016/j.aquaculture.2012.12.017

[20] Ng WK Koh CB Sudesh K: Effects of dietary organic acids on growth, nutrient digestibility and gut microflora of red hybrid tilapia, *Oreochromis* sp., and subsequent survival during a challenge test with *Streptococcus agalactiae* *Aquac Res.* 2009;40(13):1490–1500. 10.1111/j.1365-2109.2009.02249.x

[21] Da Silva BC Vieira FdN Mouriño JLP: Butyrate and propionate improve the growth performance of *Litopenaeus vannamei*. *Aquac Res.* 2016;47(2):612–623. 10.1111/are.12520

[22] Xie S Zhang L Wang D: Effects of several organic acids on the feeding behavior of *Tilapia nilotica*. *J Appl Ichthyol.* 2003;19(4):255–257. 10.1046/j.1439-0426.2003.00451.x

[23] Lim C Luckstadt C Klesius P: Use of organic acids, salts in fish diets. *Global Aquaculture Advocate.* 2010;13(5):45–46. Reference Source

[24] NRC: Nutrient requirements of warmwater fishes and shellfishes.Washington D. C.: Subcommittee on Warmwater Fish Nutrition. National Research Council. National Academies.1983 Reference Source

[25] Asriqah L Nugroho RA Aryani R: Dataset 1 in: Effect of various organic acid supplementation diets on *Clarias gariepinus* BURCHELL, 1822: Evaluation of growth, survival and feed utilization. *F1000Res.* 2018 10.5256/f1000research.15954.d216486 PMC6173120 30356366

