# Supplementary material for: Effect of various organic acid supplementation diets on Clarias gariepinus BURCHELL, 1822: Evaluation of growth, survival and feed utilization
Source: F1000Res. 2018 Sep 14;7:1465. [Version 1] doi: 10.12688/f1000research.15954.1 (PMC6173120; doi:10.12688/f1000research.15954.1)
Supplement: Peer review history

# Peer review: Referee response for version 1

**DOI:** 10.5256/f1000research.17425.r38343

**Published:** 2018-10-09

**Authors:** Indra Suharman

**Affiliations:** 1 Department of Aquaculture, Faculty of Fisheries and Marine Science, Universitas Riau, Pekanbaru, Indonesia

This papers reports on the growth performance of fish fed supplementation of organic acid in the diets of catfish. From this point of view the article is very interesting and deserves to be accepted for publication.

 The Abstract is quite clear and provide a concise conclusion of the research work. However, it is better to state the initial average weight of fish in the “Abstract”.

 The Introduction is strong supported by the literature cited and the objective of this study is clear stated.

 The Methods is quite clear and the analysis correctly explained what has been obtained from the designed work and appropriately reflects the topic studied.

 The Discussion is clear explained and the Conclusion has justified the basis of the results.

 Overall, the paper is well written and data well analyzed. I have found very interesting results as the effect of organic acid supplementation in the diets on growth performace and feed utilization of catfish.

I have read this submission. I believe that I have an appropriate level of expertise to confirm that it is of an acceptable scientific standard.

# Peer review: Referee response for version 1

**DOI:** 10.5256/f1000research.17425.r38357

**Published:** 2018-10-01

**Authors:** Ilham Alimin

**Affiliations:** 1 Feed and Nutrition Research Group, Department of Aquaculture, Jakarta Fisheries University (Sekolah Tinggi Perikanan Jakarta), Jakarta, Indonesia

Manuscript titled "Effect of various organic acid supplementation diets on *Clarias gariepinus* BURCHELL, 1822: Evaluation of growth, survival and feed utilization" could be acceptable. Study design, data processing, writing, etc. are relatively good. However, some revisions need to be made to index the manuscript.

**Abstract**

 Results:

 …were found for fish fed diets A-C and E - Kindly replace A-C with A,B,C

 …were  not affected by any types of diet - write type instead of types

 Keywords:

 …. Survival rate, …Clarias gariepinus should be in *italics*

**Introduction**

 Paragraph 2:

 Previous research stated that the use of …… - whose research? citation?

 … which help improve pepsin activity, **thus** enhancing...

 Paragraph 3:

 Since the word "generally" also means "commonly", please use one of those words

 … Previous research revealed that the values.... - whose research? citation?

 Paragraph 4:

 A strain of African catfish, *Clarias gariepinus *BRUCHELL, 1822 … - The species should be written as *C*. *gariepinus*

*….* on various feeds, such as plan material,... - material should be in plural form, please add 's'

 The role of organic acids should also be mentioned in the "Introduction" section.

**Methods**

 Site and time:

 Then, the fish had been adapted and …. - should be "Then, the fish **were** adapted and  …..

 Experimental design

 Is it true that the study was repeated four times? Or do you mean "all treatments were designed in four replicates"?

 Fish culture and feeding trial:

 Again, kindly write diets A,B,C,D and E instead of diets A-E

 Regarding feed  intake, how did you measure the feed intake? Was there any uneaten feeds?

 Measured parameters:

 Kindly replace "W0" with "Wo" and "N0" with "No"in the formula

**Results**

 When you rewrite the scientific name of the species i.e. *Clarias gariepinus*, kindly use *C*. *gariepinus*. The same concern also applicable in Discussion section. Also check it in the Table 1. 

 In Table 1 and Table 2, letter(s) ie. a,b.c should be written only when there was significant difference(s) among treatments. Therefore, FCR, FE and SR should not be highlighted with common superscript (a) since they were all statistically insignificant.

**Discussion**

 Paragraph 2:

 … and tend to be inconsistent, depend on the …. - kindly write  "depending on" instead of "depend on"

I have read this submission. I believe that I have an appropriate level of expertise to confirm that it is of an acceptable scientific standard.

# Peer review: Referee response for version 1

**DOI:** 10.5256/f1000research.17425.r38341

**Published:** 2018-09-19

**Authors:** Gitartha Kaushik

**Affiliations:** 1 Department of Zoology, North-Eastern Hill University (NEHU), Shillong, Meghalaya, India

The paper is scientifically sound in its current form and only minor, if any, improvements are suggested: 

 Kindly make these following corrections.

 1. Introduction: It is also well known that the use of antibiotics or chemical substances as a growth promoter in the feed of fish may help to improve growth, survival, and feed utilization ---- **kindly cite the article stating this statement.** 

 2. Introduction: Previous research stated that the use of non-chemical substances such as acidifiers, to increase growth performance has been performed in several fish ---- **kindly cite some previous reports**. 

 3. Introduction: Besides nutritional concern in aquafeed, generally aquaculture activities commonly produce waste, such as feed remains and feces, which can be converted into ammonia and nitrite ---- **Who stated this? Kindly cite the article** 

 4. Introduction: Clarias gariepinus BURCHELL,1822 kindly write the nomenclature following FishBase. 

https://www.fishbase.de/summary/1934  

 5. Methods: All *C. **garipienus*** were provided: **Kindly check the spelling of the species**. 

 The paper has well experimented. It can be accepted after these minor corrections.

I have read this submission. I believe that I have an appropriate level of expertise to confirm that it is of an acceptable scientific standard.
